# MicroRNAs in hematopoietic development

**DOI:** 10.1186/1471-2172-15-14

**Published:** 2014-03-31

**Authors:** Sara Montagner, Lorenzo Dehó, Silvia Monticelli

**Affiliations:** 1 Institute for Research in Biomedicine, Via Vincenzo Vela 6, Bellinzona CH-6500, Switzerland; 2 Graduate School for Cellular and Biomedical Sciences, University of Bern, Hochschulstrasse 4, Bern 3012, Switzerland

**Keywords:** Adaptive immunity, Development, Embryonic stem cells, Hematopoiesis, Hematopoietic stem cells, Innate immunity, miRNAs

## Abstract

**Background:**

MicroRNAs (miRNAs) are short non-coding RNAs involved in the posttranscriptional regulation of a wide range of biological processes. By binding to complementary sequences on target messenger RNAs, they trigger translational repression and degradation of the target, eventually resulting in reduced protein output. MiRNA-dependent regulation of protein translation is a very widespread and evolutionarily conserved mechanism of posttranscriptional control of gene expression. Accordingly, a high proportion of mammalian genes are likely to be regulated by miRNAs. In the hematopoietic system, both transcriptional and posttranscriptional regulation of gene expression ensure proper differentiation and function of stem cells, committed progenitors as well as mature cells.

**Results:**

In recent years, miRNA expression profiling of various cell types in the hematopoietic system, as well as gene-targeting approaches to assess the function of individual miRNAs, revealed the importance of this type of regulation in the development of both innate and acquired immunity.

**Conclusions:**

We discuss the general role of miRNA biogenesis in the development of hematopoietic cells, as well as specific functions of individual miRNAs in stem cells as well as in mature immune cells.

## Review

### Introduction

The differentiation and homeostasis of the hematopoietic system requires complex and interconnected molecular networks that need to be carefully regulated. In fact, a wide repertoire of different immune cells originates from hematopoietic stem cells (HSCs) in the bone marrow, cells characterized by multipotency and self-renewal capabilities. These cells generate the whole range of mature cells in the blood, whose functions also need to be tightly controlled in order to avoid cellular responses from running amok and becoming damaging for the organism. Both transcriptional and posttranscriptional regulatory mechanisms ensure proper cellular differentiation and function through modulation of cell death, proliferation, activation and lineage commitment. Among the regulatory factors involved in these processes are microRNAs (miRNAs), a class of short non-coding RNAs whose pivotal roles in fine-tuning the hematopoietic system have emerged in the past several years. The first groundbreaking study about a role for miRNAs in the differentiation of the immune system showed how forced expression of miR-181 in hematopoietic progenitors markedly increased the number of B lymphocytes, with a concomitant reduction of T lymphocytes [1]. Since then, the role of miRNAs in the immune system has been extensively studied and these molecules were discovered to be critical regulators of both normal immune functions as well as disease. Highlighting the widespread conservation of this type of regulation of gene expression, the miRBase database (http://www.mirbase.org) lists a constantly increasing number of miRNAs expressed in more than 200 different species [2]. In this review we will provide a snapshot on the role of miRNAs specifically during the development of immune cells from hematopoietic progenitors.

### MicroRNAs

MiRNAs are small non-coding RNAs, usually 21–25 nucleotides (nt) long, present in a wide variety of organisms and able to regulate gene expression by targeting messenger RNAs (mRNAs). Specifically, they act by binding to imperfect sites in the 3′ untranslated region (UTR) of a target mRNA, resulting in repression of translation and/or degradation of the targeted molecule, either of which leads eventually to gene silencing. MiRNA biogenesis involves several steps: a long primary transcript (pri-miRNA) is transcribed by a RNA polymerase (usually RNA polymerase II) and is typically polyadenylated. The pri-miRNA may contain one or more hairpins, as miRNAs can be clustered together to give rise to polycistronic transcriptional units [3]. In the nucleus, the hairpin is excised from the pri-miRNA by the Microprocessor complex, minimally composed by the RNAseIII Drosha and the double-stranded RNA-binding protein DGCR8. The resulting hairpin precursor (pre-miRNA) is usually 60–110 nt long, and is actively exported to the cytoplasm by the nuclear-membrane protein Exportin-5 [3]. In the cytoplasm, another protein complex containing the RNAseIII enzyme Dicer further processes the pre-miRNA to the mature miRNA, which is loaded onto the RNA Induced Silencing Complex (RISC). Only one of the two strands of the original pre-miRNA stem remains bound to the RISC (the guide strand) as mature miRNA, whereas the other strand (passenger strand or miRNA*) may be eliminated. Importantly, Drosha processing of the pri- to pre-miRNA can be a cotranscriptional process that does not impair splicing of the primary transcript [4,5]. This is particularly important for those miRNAs that are transcribed within introns of protein-coding genes, as miRNA processing does not impair mRNA maturation, and protein synthesis is therefore not affected by Drosha cleavage [5]. Besides the canonical biogenesis of miRNAs outlined above, a small portion of miRNA precursors named mirtrons derive from spliced introns and do not require Drosha activity for their maturation [6,7]. Once generated, the RISC-miRNA complex recognizes its cognate binding sites in the 3′UTR of target mRNAs to induce gene silencing.

### MicroRNAs in embryonic stem cells

Recent studies elucidated the role of miRNAs in the regulation of the biological function of embryonic stem (ES) cells, derived from the inner cell mass of blastocysts. ES cells are characterized by self-renewal capability and totipotency, features that allow these cells to maintain their own identity throughout mitotic cell division and to differentiate into the entire range of specialized cell types required in a multicellular organism. Totipotency is ensured by the expression of few transcription factors such as Nanog, Oct4 and Sox2 [8-10]. Indeed, ectopic expression of some of these factors (Oct3/4, Sox2, c-Myc, and Klf4) is enough to reprogram differentiated cells to a totipotent state [11]. A strong downregulation of these markers together with the expression of lineage-specific activators trigger ES cell differentiation in the three primary germ layers (endoderm, ectoderm and mesoderm). Importantly, the transcriptional regulation of ES cell differentiation has been also associated with changes in the miRNA expression profile [12-14]. The first evidence that miRNAs were involved in regulating ES cell biology came from studies in which miRNA biogenesis was eliminated through deletion of the gene encoding for Dicer (*Dicer1*). Genetic ablation of *Dicer1* in mouse ES cells resulted in morphological abnormalities in the early stages of development and embryonic lethality [15]. Consistently, Dicer-deficient ES cells expressed ES cell-specific markers such as the short α6-*integrin* isoform and *Oct4*, but failed to generate detectable teratomas upon subcutaneous injection into nude mice, and did not express any of the most common differentiation markers for both the mesodermal and endodermal lineages, such as *T-Brachyury*, *Gata1*, *Bmp4* and *Hnf4*, indicating that Dicer ablation leads to defects in ES cell differentiation [16]. Another important level of miRNA regulation in ES cells is related to epigenetic modifications in the genome. Indeed, a global reduction of DNA methylation was observed in Dicer-deficient ES cells, which was associated with reduced centromeric silencing and affected telomere-length homeostasis [16,17]. Moreover, reduced levels of *de novo* methylation in ES cells in the absence of Dicer were associated to downregulation of DNA methyltransferases with a mechanism that was dependent on the expression of the miR-290 miRNA cluster [17]. Consistently, the defects in DNA methylation could be rescued by transfection of the miR-290 cluster in Dicer-deficient ES cells, indicating that *de novo* DNA methylation in ES cells is at least in part controlled by miRNAs [18]. Another indication that miRNAs have a crucial role in ES cell biology came from studies performed in the absence of the *Dgcr8* gene, which encodes for an essential component of the nuclear Microprocessor complex [19]. Specifically, ES cells deficient for DGCR8 lacked miRNA expression, and during differentiation the main pluripotency markers *Oct4, Sox2* and *Nanog* could only partially be silenced. Moreover, cells were severely impaired in their ability to express differentiation markers for the three germ layers including *Fgf5*, *T-Brachyury* and *Hnf4a*. In particular, *Fgf5*, a marker of primitive ectoderm that was highly expressed in wild-type embryoid bodies (EBs) from day 2 of differentiation, could be detected only at day 8 in *Dgcr8*-deleted EBs and never reached the levels of the control. Moreover, a block in the G1-S transition during cell cycle progression was observed in *Dgcr8*-deleted ES cells, and after injection in immunocompromised mice these ES cells developed undifferentiated teratomas, therefore confirming that miRNAs are indeed fundamental for proper differentiation of ES cells [19].

In the last decade, a role for miRNAs has become evident not only in the maintenance of the “stemness” and in the early induction of differentiation through modulation of the expression of the master pluripotency genes, but also during early organogenesis. For example, using both gain- and loss-of-function approaches, Krichevsky *et al*. demonstrated that the early overexpression of miR-124a in neural precursors prevented gliogenesis, whereas miR-9 favored differentiation into neurons [13]. Inhibition of miR-9 in neural precursors led to reduced neuronal differentiation through increased STAT3 phosphorylation, which is known to be involved in the inhibition of neuronal differentiation [20-22]. On the contrary, the concomitant overexpression of both miR-124a and miR-9 repressed induction of the STAT3 pathway preventing differentiation of the neural precursors into astrocytes [13]. The small C-terminal domain phosphatase 1 (SCP1) was also identified as a primary target for miR-124a in neurons, and SCP1 repression mediated by miR-124 induced neuronal differentiation, pointing towards a crucial role for this miRNA in regulating signaling events that lead to neuron development [23]. In another example of miRNA-dependent regulation of differentiation to specific lineages, a role for miR-1 and miR-133 was established for differentiation to cardiomyocytes: both of these miRNAs were enriched in cardiomyocytes derived from ES cells at the early stages of cardiac mesoderm selection, but they were repressed in the ectodermal and endodermal lineages [14]. However, these two miRNAs showed opposing behaviors during further differentiation into the muscle lineage, since miR-1 promoted and miR-133 blocked the differentiation into cardiac progenitors or skeletal myoblasts [14]. Confirming the fact that miR-1 is an essential regulator of cardiogenesis, deletion of miR-1-2 in mice revealed dysfunctions in cardiac morphogenesis, electrical conduction and cell cycle control [24].

### Hematopoietic differentiation

**Figure 1:**
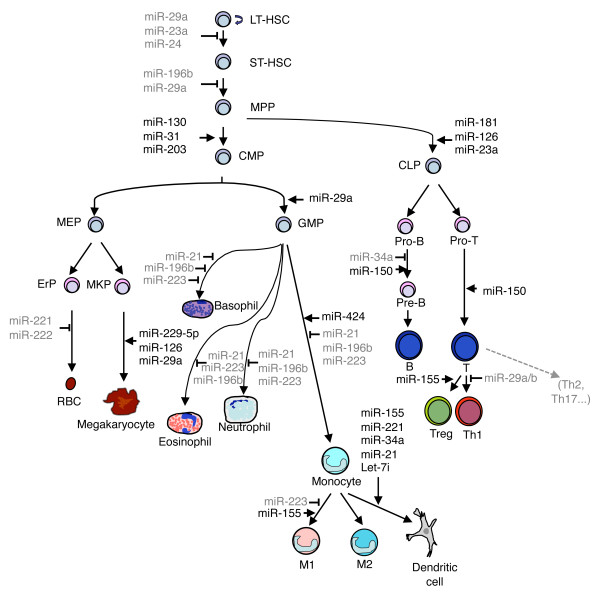
**Schematic representation of the hematopoietic system with some of the miRNAs involved in its regulation.** MiRNAs that block a specific stage are indicated in grey, whereas miRNAs that promote development are indicated in black. LT-HSC: long-term hematopoietic stem cell; ST-HSC: short-term hematopoietic stem cell; MPP: multipotent progenitors; CMP: common myeloid progenitor; CLP: common lymphoid progenitor; MEP: megakaryocyte-erythroid progenitor; GMP: granulocyte-macrophage progenitor; ErP: erythroid progenitor; MKP: megakaryocyte progenitor; RBC: red blood cells.

A properly regulated hematopoietic differentiation is essential throughout the life of an organism to form all blood cells required for gas exchange, wound healing and for an appropriate defense from invading pathogens. During embryonic development, hematopoiesis takes place at first in the yolk sac, then the placenta and major arteries become also involved, followed by the fetal liver and finally the bone marrow [25,26]. In the adult, bone marrow-resident stem cells can be mainly subdivided into two defined subsets: long-term reconstituting HSCs (LT-HSCs) and short-term reconstituting HSCs (ST-HSCs). LT-HSCs maintain their self-renewal and multi-lineage differentiation potential throughout the entire life of the organism and give rise to ST-HSCs, which are instead more limited in terms of self-renewal capability, although they maintain multipotency and are able to differentiate into multipotent progenitors (MPPs) [27] (Figure 1). Although a few different markers can identify various subpopulations of stem cells, for the most part the cell hierarchies within the HSC population can be defined by the differential expression of the following surface markers: Sca-1, c-Kit, CD135, CD48, CD150 and CD34. LT-HSCs are characterized by the total absence of lineage specific markers on the surface (“lineage negative” or Lin– cells), and by the expression of Sca-1, c-Kit and CD150. When LT-HSCs differentiate into ST-HSCs, they progressively express CD34, followed by CD48 expression in MPPs [28]. Distinct populations of MPPs generate lineage-committed oligopotent progenitors including the common lymphoid progenitor (CLP), common myeloid progenitor (CMP), megakaryocyte-erytrocyte progenitor (MEP) and granulocyte-monocyte progenitor (GMP). Lineage committed progenitors, in turn, give rise to mature cells including B, T and NK cells, granulocytes, monocyte/macrophages, mast cells and megakaryocytes. Flow cytometric analysis allows the discrimination of the myeloid and lymphoid lineages based on the downregulation of CD150 and the acquisition of CD135 markers, respectively [28]. Overall, the entire hematopoietic system is organized as a tree where the developmental potential is restricted in each branch point: each step is tightly regulated by environmental signals from the niche, signaling molecules, specific transcription factors as well as miRNAs [27]. Representative examples of miRNAs involved in hematopoietic differentiation are discussed in the following paragraphs, and are summarized in Figure 1.

### The role of miRNAs in maintaining stemness and favor HSC differentiation

The importance of miRNAs in regulating the state of HSCs in the hematopoietic system was shown upon ablation of Dicer specifically in the hematopoietic stem/progenitor compartment, which resulted in loss of functional HSCs due to increased apoptosis [29]. Interestingly, a single miRNA, miR-125a, was found to be sufficient to modulate HSC self-renewal and numbers, and to protect Lin– progenitor cells from apoptosis [29]. Similarly to what has been described for ES cells, different families of miRNAs regulate HSC self-renewal and differentiation capabilities, and HSCs are characterized by a specific miRNA signature in each state of differentiation. For example, LT-HSCs showed enriched expression of a group of miRNAs that included miR-125a, miR-125b, miR-155, miR-99a, miR-126, miR-196b, miR-130a, miR-542-5p, miR-181c, miR-193b and let7e [30]. In order to assess their impact on long-term hematopoietic engraftment, these miRNAs were overexpressed through retroviral transduction in bone marrow cells and injected into lethally irradiated recipients. Depending on the miRNA overexpressed, different phenotypes emerged in the hematopoietic compartment. In particular, miR-125b-5p, miR-126-3p and miR-155 conferred a competitive advantage to the engrafted bone marrow, whereas miR-196b, miR-181c, let7e and miR-542-5p caused a significant disadvantage. In all cases, the effects of the engraftment were observed on all downstream lineages, suggesting that these miRNAs regulate stem cell homeostasis rather than influencing differentiation to any particular phenotype. Importantly, many of the miRNAs that were identified in this study have also been shown to be dysregulated in acute myeloid leukemia (AML). For example, miR-125b expression in bone marrow cells led to a myeloproliferative disorder that could progress to aggressive myeloid leukemia [30]. Along the same line, miR-196b was shown to be overexpressed specifically in the majority of patients with mixed lineage leukemia (MLL), but not in other types of leukemia [31]. Moreover, with the exception of miR-193b, all of the miRNAs that were shown to be enriched in mouse HSCs also showed significant enrichment in CD34+ progenitors derived from human cord blood compared with mature CD34− cells [30]. Early human hematopoietic cells are characterized by high expression levels of the CD133 surface marker, and can generate CD34+ progenitors, which in turn can differentiate to all hematopoietic lineages [32,33]. Human CD133+ cells were analyzed in a combined miRNA-mRNA expression profile to identify miRNAs that might contribute to the maintenance of the HSC ‘stemness’ [34]. Among the miRNAs identified, target analysis revealed that miR-29a downregulated the expression of the actin binding protein TMP1, as well as of FZD5, a receptor acting in the Wnt-signaling pathway [34]. Interestingly, MiR-29a was also shown to be involved in the regulation of early hematopoiesis and myeloid commitment in the mouse system. MiR-29a was highly expressed in early progenitors where it likely contributed to the maintenance of the undifferentiated status, while it was downregulated during differentiation [35]. Accordingly, ectopic expression of miR-29a in mouse HSCs resulted in the acquisition of self-renewal capabilities of myeloid precursors, biased myelopoiesis and development of a myeloproliferative disorder that could progress to AML. Highlighting its importance as a regulator of self-renewal and proper myelopoiesis, not only miR-29a overexpression in the mouse induced an AML-like disease, but miR-29a was also found strongly overexpressed in patients with AML [35].

While most of the miRNAs discussed so far were mainly found to be involved in somehow maintaining the self-renewal feature typical of stem cells, other miRNAs instead favored HSC differentiation to more committed cell types. MiRNA expression profiles from human CD34+ hematopoietic progenitors and *in vitro* differentiated erythroblasts, megakaryoblasts, monoblasts and myeloblast precursors showed a specific upregulation of miR-299-5p in megakaryoblasts together with the downregulation of its putative target genes [36]. Accordingly, forced expression or inhibition of miR-299-5p in CD34+ cells altered the colony formation capacity of these cells, modulating megakaryocytic-granulocytic differentiation *versus* erythroid-monocytic commitment [36]. MiR-221 and miR-222 were also involved in the regulation of both the early and late stages of erythropoiesis: CD34+ cells transfected with either miRNA mimics or lentiviruses to force expression of these two miRNAs showed inhibition of proliferation and accelerated differentiation to the erythropoietic lineage through downregulation of one of their target, c-Kit [37]. In order to provide a more complete scenario of the regulation of hematopoietic differentiation by miRNAs, Petriv *et al.* developed a high-throughput technique that combined microfluidics with the sensitivity and specificity of qRT-PCR, allowing miRNA profiling on limited cell numbers or even at a single cell level [38]. Using this technique the authors were able to analyze 27 different cell populations, including ES cells, early hematopoietic precursors, CMP, CLP, GMP, MEP as well as mature cells representative of all lineages. Interestingly, reconstruction of a hematopoietic hierarchical tree based on the miRNA expression pattern grouped the 27 cell types investigated into six main branches: stem cells and multipotent progenitors, lymphoid cells, and finally four different major branches of myeloid cells. Several miRNAs were then found to change in expression at distinct nodes. In particular, the most differentially expressed miRNAs between stem cells and progenitors relative to the more mature populations included miR-125b, miR-196a/b, miR-130a, let-7d, miR-148b and miR-351. Similarly, changes in expression of a number of other miRNAs distinguished CMPs from CLPs or CMPs from more committed progenitors [38]. Despite the fact that a number of miRNAs have been described to be specifically expressed in various steps during the differentiation process of early hematopoietic progenitors, the targets that are regulated by these miRNAs and their overall biological impact on lineage commitment and HSCs homeostasis remain for the most part to be uncovered, and further analysis will be required to completely dissect the molecular network regulated by miRNAs in hematopoietic differentiation.

### Innate immunity

The innate immune system is the first responder towards invading pathogens. Innate immune cells include dendritic cells, granulocytes, monocytes/macrophages and mast cells, which are all bone marrow-derived. Granulocytes are short-lived cells that include neutrophils, basophils and eosinophils, which can be distinguished by their staining properties, surface markers and functions. MiRNAs have been shown to regulate a number of aspects of innate cell differentiation and function as well as of innate responses (for a recent review on the topic see [39]). A key miRNA that was shown to regulate granulocytic differentiation and function is miR-223, which showed a pattern of expression highly lineage-specific. Indeed, miR-223 was shown to be expressed at low levels in HSCs and CMPs, and to be steadily upregulated during differentiation to granulocytes and repressed during differentiation to the alternative monocytic fate [40]. Interestingly, lack of miR-223 in mice resulted in an expanded granulocytic compartment due to an increased number of progenitors. The transcription factor Mef2c was shown to be a target for miR-223 in these mice, and genetic ablation of this transcription factor corrected the phenotype induced by the absence of miR-223. In addition, granulocytes that developed in *miR-223*-deleted animals showed terminal maturation and increased sensitivity to activating stimuli, to the point that mice developed neutrophil-mediated disease with spontaneous lung pathology and excessive tissue damage after challenge [40]. Of note, expression of miR-223 in human acute promyelocytic leukemia cells led to enhanced differentiation in response to treatment with retinoic acids [41,42]. Despite some differences in findings that can be due at least in part to the different systems used, miR-223 has undoubtedly emerged as an important regulator of both the generation and function of granulocytic cells, by modulating their differentiation as well as their sensitivity to activating stimuli [40]. Highlighting the overall importance of the miRNA pathway in regulating myelopoiesis, deletion of Dicer specifically in myeloid-committed progenitors led to defective myeloid development, with reduced differentiation of these cells and regained self-renewal potential [43].

Macrophages are long-lived, tissue-resident cells derived from circulating monocytes. They are remarkable phagocytic cells committed to the engulfment and elimination of invading microorganisms, and they can also contribute to the activation of the adaptive immune response by releasing inflammatory and signaling molecules. MiR-17-5p, miR-20a and miR-106a were all implicated in the differentiation of monocytes. Specifically, these miRNAs were shown to act in a molecular circuitry with the transcription factors AML1 (Runx1) to regulate expression of the macrophage-colony stimulating factor receptor (M-CSFR) and consequently modulating monocytic differentiation [44]. The transcription factor AML1 is crucial for the development of the hematopoietic system, as shown by the fact that deletion of the *Aml1* gene in mice is embryonically lethal due to failed hematopoiesis [45]. During monocytic differentiation, AML1 was shown to be upregulated at the protein but not mRNA level, concomitantly with declined expression of miR-17-5p, miR-20a and miR-106a [44]. Reduced expression of these miRNAs unblocked translation of AML1, which in turn could inhibit transcription of these miRNAs in a negative feedback loop, finally allowing cell differentiation [44]. The importance of carefully tuning AML1 expression is also highlighted by the fact that in myeloid leukemias the fusion protein AML1-ETO results in altered transcriptional activity of AML1 [46-50], leading to the expansion of immature cells in a scenario that resembles overexpression of miR-17-5p, miR-20a and miR-106a [44]. Highlighting the importance of the miR-17 ~92 cluster in cancer and leukemia, a number of studies have shown that this miRNA cluster (consisting of miR-17-5p, miR-18a, miR-19a, miR-19b, miR-20a, and miR-92a) is frequently overexpressed in several solid and lymphoid malignancies [51], and forced expression of this locus led to lymphoproliferation [52-54]. Interestingly, deletion of the miR-17 ~92 cluster is associated with altered embryonic development and death [55], and dyregulation of a number of targets of this cluster (including the tumor suppressor PTEN and the proapoptotic protein Bim [52]) is thought to contribute to tumorigenesis.

Several miRNAs are involved not only in monocytic differentiation but also in macrophage polarization. Macrophages can be sub-divided into different phenotypically distinct subpopulations: two of these populations include classically activated or M1 and alternatively activated or M2 macrophages, which are characterized by distinct surface markers, cytokine production capabilities, differentiation requirements and functional properties [56]. One of the miRNAs shown to be potentially involved in regulating the balance between M1 and M2 differentiation is miR-155 [57]. MiR-155 was shown to be upregulated in macrophages upon LPS treatment [58], and in human monocytes, it was shown to directly target IL-13Rα1, the receptor for IL-13, a critical cytokine for the balance between M1 and M2 macrophage polarization [57]. MiR-155 expression led to reduced IL-13-dependent STAT6 phosphorylation and changed the mRNA expression profile of several IL-13/STAT6-dependent genes. Along the same line, IL-10, which is an anti-inflammatory cytokine also known to be important in regulating M1/M2 polarization, was shown to inhibit LPS-dependent upregulation of miR-155, further suggesting a role for this miRNA in the modulation of the balance between differently polarized macrophages and more in general in the regulation of inflammation [56,59].

Dendritic cells (DC) are the most specialized and potent antigen presenting cells (APCs) of the immune system, essential to induce adaptive immunity. These tissue-resident cells can be subdivided in different subsets, including conventional DCs (cDC) and plasmacytoid DCs (pDCs). The expression of miRNAs during DC differentiation from monocytes was extensively studied using microarray analysis [60-62]. For example, Lu *et al.* analyzed the miRNA profile during development of immature (imDC) and mature DCs from monocytes [62]. Specifically, miR-155 and miR-221 regulated human DC differentiation by modulating proliferation and cell death. MiR-221 and miR-155 expression was relatively low in monocytes, which expressed higher levels of the cell-cycle inhibitor p27^Kip1^. Upon induction of differentiation, miR-221 was upregulated with a concomitant reduction of its target p27^Kip1^, driving DC maturation. During maturation DCs downregulated miR-221 and upregulated miR-155, resulting in accumulation of p27^Kip1^ and induction of IL12p70, contributing to the regulation of apoptosis and promoting terminal differentiation [62]. MiR-221 and miR-222 were also shown to be expressed at higher levels in cDCs compared to pDCs, and inhibition of these miRNAs decreased the number of cDCs and favored pDCs accumulation [63].

MiR-155 is another miRNA important for DC functions: it is contained within the noncoding B cell integration cluster (*bic*) gene, and its importance in DC differentiation and function is highlighted by the fact that DCs lacking *bic*/*miR-155* exhibited reduced functionality, with a strong impairment in their ability to stimulate T cells [64]. As for the mechanism of action of miR-155, microarray and functional experiments revealed that one of the key roles of miR-155 in DCs is the silencing of the transcription factor c-Fos, and cells with dysregulated c-Fos expression phenocopied the lack of miR-155 [65]. Interestingly, a role for the less abundant passenger strand of miR-155, miR-155*, was also identified for pDC activation upon TLR7 stimulation, and although miR-155 and miR-155* are processed from a single precursor, they appeared to have opposing effects on the production of type I interferons from pDCs [66].

### Adaptive immunity: B cell maturation and germinal center responses

The adaptive immune system is composed by highly specialized cells (B and T lymphocytes) that are able to recognize specific antigens, generate responses to eliminate pathogens and finally develop an immunological memory that can quickly respond to a subsequent invasion from the same pathogen. B lymphocytes respond to antigen recognition by producing a wide spectrum of antibodies. The molecular network that leads to proper B cell development must be finely orchestrated, and includes miRNA-dependent posttranscriptional regulation. Indeed, the overall impairment of miRNA biogenesis induced by *Dicer* ablation in B cell precursors caused a block of B cell differentiation in the transition from pro- to pre-B cells [67]. Gene expression profiles of *Dicer*-deficient pro-B cells revealed a specific miR-17 ~92 signature in the 3′UTRs of the upregulated genes, highlighting the importance of this particular cluster of miRNAs in B cell differentiation [67]. Indeed, mice in which miR-17 ~92 were overexpressed developed spontaneous lymphoproliferative disease and autoimmunity [52]. Not only the expression of miR-17 ~92 is important to regulate survival of early B cell progenitors in a cell-autonomous manner, but these miRNAs also regulate B cell responses by controlling differentiation of follicular helper T lymphocytes (T_FH_). This subset of T helper lymphocytes is crucial to provide signals to B cells to develop appropriate antibody responses, and genetic deletion of miR-17 ~92 led to substantially compromised differentiation of T_FH_ cells and consequently to reduced antibody responses to infection [68,69].

Consistent with a crucial role of miRNAs in regulating B cell functions, the formation of germinal centers in secondary lymphoid organs was drastically compromised in the absence of Dicer: *Dicer1*^*fl/fl*^*Aicda*^*Cre/+*^ mice, in which Dicer ablation is under the control of the activation-induced cytidine deaminase (AID) promoter and therefore occurred only in activated B cells, showed impaired class-switch recombination as well as compromised B cell memory formation [70]. In addition, the selective ablation of another fundamental component of the RISC complex, Argonaute 2, affected pre-B cell differentiation as well as maturation of erythroid precursors [71]. Taken together, these studies demonstrated the critical role of Dicer and miRNAs in B cell differentiation both in the bone marrow and in the periphery.

As for the role of individual miRNAs in B cell differentiation and function, one example is provided by miR-150 that is normally expressed at low levels in HSCs, and is instead upregulated during differentiation. Its ectopic expression in HSCs was associated with a severe impairment in the formation of mature B cells and with the block of the transition from the pro- to pre-B stage [72]. In particular, B cell progenitors deleted for miR-150 showed impaired B cell responses and differentiation defects due to the dysregulation of c-Myb, a target for miR-150 known to play a critical role in multiple steps of lymphocyte development [73]. The pro- to pre-B cell transition is also regulated by miR-34a through inhibition of the transcription factor Foxp1 [74]. The constitutive expression of miR-34a induced a partial block in B cell development while its knockdown resulted in increased number of mature B cells in the bone marrow, and knockdown of Foxp1 induced a phenotype that resembled the one observed upon overexpression of miR-34a [74]. Additionally, miR-34a itself was shown to be induced at a transcriptional level by the transcription factor p53, and accordingly, mice in which *p53* was deleted showed accumulation of pro-B cells [75-78]. p53 may therefore regulate B cell development at least in part through the upregulation of miR-34a and consequent inhibition of Foxp1 [74]. Finally, further studies demonstrated the involvement of miRNAs also in the latest stages of B cell maturation. Experiments using genetic ablation and transgenic approaches to analyze the effect of altered miR-155 expression revealed impaired germinal center reactions in the context of T cell-dependent antibody responses as well as altered cytokine production [64,79]. Moreover, B cells lacking miR-155 generated reduced extrafollicular and germinal centre responses upon stimulation with both thymus-dependent and -independent antigens, which correlated with the reduced number of rearranged IgG antibodies [80]. Interestingly, miR-155–deficient B cells showed upregulation of the transcription factor PU.1, whereas PU.1 overexpression in wild-type B cells resulted in reduction of IgG1-switched cells, suggesting that miR-155 directly regulates class-switching recombination in secondary lymphoid organs through PU.1 expression [80]. AID was also shown to be a target for miR-155 in B cells [81,82]. AID is required for immunoglobulin gene diversification through somatic hypermutation (SHM) and class-switch recombination (CSR), and disruption of miR-155–dependent AID regulation led to quantitative and temporal dysregulation of AID expression resulting in altered affinity maturation and CSR, but also in a high degree of chromosomal translocations [81,82]. Taken together these results implicate posttranscriptional regulation of gene expression mediated by miRNAs in all stages of B cell differentiation, activation and memory formation.

### Adaptive immunity: T cell differentiation and polarization

The stages of development of T lymphocytes into functional cells are characterized by the expression not only of the T cell receptor (TCR) and associated molecules, which allow antigen recognition and signal transduction within the cell, but also by the expression of the coreceptor molecules CD4 and CD8. CD4 and CD8 double-negative (DN) cells are the early T cell progenitors, which differentiate into CD4/CD8 double-positive (DP) cells and then in CD4 or CD8 single-positive (SP) cells. Based on the expression of the surface markers CD44 and CD25, DN thymocytes can be further divided into four stages (DN1-4) following the order of appearance during differentiation. In order to investigate the biological function of the miRNA pathway in T lymphocyte development, the selective ablation of the *Dicer1* gene in DP thymocytes was performed using a Cre recombinase under the control of the CD4 promoter [83]. Upon *Dicer1* deletion, a severe block in the development of peripheral CD8 T cells was observed, as well as a smaller, but still significant, decrease of CD4 T cells in the spleen and lymph nodes. Further characterization of these CD4 T lymphocytes showed increased levels of apoptosis, defective proliferation and aberrant cytokine production, indicating that miRNAs are essential for the proper maturation and function of T lymphocytes in the periphery [83]. Importantly, when *Dicer1* was selectively ablated earlier during thymocyte development at the DN3 stage using an lck-Cre transgene, numbers of αβ T cells were reduced, correlating with an increased susceptibility to cell death of these cells, while the CD4/CD8 lineage choice appeared largely intact [84]. While Dicer was shown to regulate centromeric silencing, affecting telomere length homeostasis as well as *de novo* methylation in ES cells [16], conditional ablation of *Dicer1* in DN3-stage thymocytes did not appear to be required for epigenetic regulation of these cells. Indeed, the transcriptional silencing at pericentromeric satellite sequences, DNA methylation and X chromosome inactivation in female cells, as well as the stable silencing of the *Tdt* gene, which is involved in TCR rearrangement, were globally not affected in *Dicer1*-deleted thymocytes, indicating that the epigenetic marks established during differentiation of T cells are maintained independently from Dicer expression [84].

Since Dicer was shown to be involved in several aspects of the hematopoietic differentiation as well as lymphocyte development, it is not surprising that the overall impairment of the miRNA biogenesis could interfere also with CD4 T helper (Th) cell polarization, as well as with the differentiation of regulatory T cells (Tregs) (see Refs. [85,86] for recent reviews on the topic). Gene-deleted mouse models of either Drosha or Dicer in the T cell compartment resulted in spontaneous T cell activation, inflammatory disease and premature death due to compromised Treg differentiation and reduced suppressive activity, recapitulating the phenotypes observed in the absence of Foxp3 [87,88]. Importantly, Dicer and Drosha deficiencies were also shown to alter T lymphocytes polarization. Th1 cells are characterized by strong IFN-γ production, whereas Th2 responses are mainly based on IL-4 production. Dicer-deficient CD4 T cells could not repress IFN-γ production and continued to produce IFN-γ even after two consecutive rounds of activation under Th2 conditions, indicating that Dicer is required for repression of the Th1 genetic program [83]. In addition, the vast majority of Dicer-deficient Th2 cells restimulated and further cultured under Th1 polarizing conditions regained IFN-γ production, further indicating that Dicer is required to repress the Th1 genetic program and also suggesting that Dicer deficiency may impair stable Th2 commitment [83]. Importantly, a similar phenotype was observed in Drosha-deficient as well as in DGCR8-deficient T cells [87,89], indicating that the lack of miRNAs in T cells determines a bias towards IFN-γ production and reduced proliferation and survival after stimulation [89]. In order to identify individual miRNAs primarily responsible for these observed phenotypes, miRNAs were individually screened for their capacity to correct aberrant IFN-γ production in the absence of DGCR8 [89]. Using this method, miR-29a and miR-29b were identified as suppressors of the Th1 fate. In particular, transfection of both miR-29a and miR-29b in DGCR8-deficient T cells induced a reduction in IFN-γ production in a dose-dependent manner and the same phenotype was observed even in conditions that strongly promoted Th1 cell differentiation. Interestingly, these miRNAs had no significant effect on the total frequency of IL-4– or IL-2–producing cells and they did not affect cell proliferation or viability, indicating that the effect of miR-29a and miR-29b goes through specific regulation of IFN-γ production. Indeed, both T-bet and Eomes, transcription factors critical for IFN-γ production, were identified as direct targets of miR-29a/b [89]. Along the same line, infection of wild-type mice with intracellular bacteria induced downregulation of miR-29a/b in CD4 and CD8 T cells, with enhanced IFN-γ production and consequent elimination of the pathogen [90]. Transgenic mice engeneered to express a miR-29 ‘sponge’ that competed with endogenous miR-29 targets, showed enhanced Th1 responses and greater resistance to infection [90].

In a hallmark example of a role of miRNAs in regulating TCR signaling, Li *et al.* identified miR-181a as an intrinsic modulator of T cell sensitivity to peptide antigens [91]. MiR-181a was shown to be expressed at high levels in the earliest stages of T cell differentiation, namely in DN1-3 cell populations, and to drop dramatically in DN4 cells, as well as in DP and SP thymocytes, indicating that this miRNA may play a role in T cell maturation [91]. Further experiments demonstrated that miR-181a regulates thymic positive and negative selection through the modulation of TCR signaling strength, at least in part by downregulating a number of phosphatases, and therefore leading to elevated levels of phosphorylated signaling proteins and to an overall reduction of the TCR activation threshold [91]. Indeed, the TCR strength of signal is regulated by the phosphorylation state of several kinases that establish an “excitation threshold” for T cell activation. The tyrosine phosphatases SHP-2, PTPN22 and the ERK-specific phosphatases DUSP5 and DUSP6 were all shown to contain miR-181a binding sites and to be specifically repressed by miR-181a. As a consequence, T cells overexpressing miR-181a showed significantly higher basal levels of phosphorylated ERK (pERK) compared to control cells, with both the maximum peak of ERK phosphorylation and the kinetic of dephosphorylation being altered in presence of miR-181a. These results indicate that miR-181a expression is important to maintain high levels of pERK through inhibition of phosphatases, thus reducing the activation threshold to peptide antigens [91].

In the final stages of T lymphocyte maturation, some of the activated cells acquire a long-lived memory phenotype that allows a more rapid and effective response to pathogens that have been previously encountered. MiR-146a was shown to be induced upon TCR stimulation to levels that directly correlated with the strength of TCR signaling [92]. Importantly, this miRNA was shown to be involved in effector and memory CD8 responses, with mice lacking miR-146a responding more vigorously and persistently to peptide antigens, in line with a role for this miRNA as a general negative regulator of the immune response, but possibly also as a modulator of the TCR signaling threshold and memory formation [92-94]. It would be tempting to speculate that, resembling the effect of miR-181a in regulating the TCR signal strength in thymocytes, miR-146a may have a similar role in the periphery by modulating the activation of NF-kB upon TCR recognition of the antigen. Overall, although work remains to be done to elucidate the details of regulation, data from the past several years leave no doubts about an important role for miRNAs in T cell development, differentiation to the appropriate subsets, establishment of immunological memory and the maintenance of homeostasis in the periphery [86].

## Conclusions

MiRNAs are fundamental regulators of hematopoiesis, critically implicated in regulating both the maintenance of the ‘stemness’ of the early progenitors and the various stages of differentiation to mature cells. While their overall role in these processes is quite established, we still need to elucidate the function of individual miRNAs in distinct cell types or developmental stages, especially since the effect of a given miRNA may be exquisitely dependent on the cellular context in which it is expressed. Indeed, since what eventually matters for the regulation of a miRNA target is the concentration of the given miRNA relative to that of its target, factors like miRNA abundance and stability, ability to participate in multiple rounds of targeting, as well as expression of cell-specific mRNA targets, will all influence miRNA networks and miRNA-transcription factor circuits (discussed in [95]). Depending on all of the abovementioned factors, one could easily envision situations in which the same miRNA may act on different targets and lead to different outcomes in different cellular contexts. Moreover, miRNAs emerged as an important component of the many checks and balances required to keep the immune system from getting out of control, potentially leading to tissue damage and disease. Indeed, the importance of miRNAs in these processes is emphasized by their aberrant expression in pathologic conditions such as autoimmune disease and cancer [96-100]. Since a chronic dysregulation of miRNA expression may lead to aberrant differentiation, uncontrolled proliferation and break of immunological tolerance, understanding miRNA-mediated control of gene expression could provide innovative strategies for treatment of hematopoietic malignancies and other diseases. In one potentially useful therapeutic application of miRNAs, miR-150 was identified as a miRNA responsible for reduced hematopoietic recovery following chemotherapy-induced suppression of peripheral blood parameters [101]. These data further highlights how manipulation of miRNA expression may become important in the future in a variety of clinical and more translational settings.

## Competing interests

The authors have no competing interests to declare.

## Authors’ contributions

All authors contributed to conceive and write this manuscript. All authors read and approved the final manuscript.
